# Accelerating the Reaction Kinetics of Na_2_CO_3_-Activated Slag Mortars by Calcined Recycled Concrete Fines

**DOI:** 10.3390/ma15155375

**Published:** 2022-08-04

**Authors:** Hao Wang, Liang Wang, Ying Xu, Ke Cao, Yan Ge, Xuepeng Wang, Qi Li

**Affiliations:** 1 School of Civil Engineering and Architecture, Anhui University of Science and Technology, Huainan 232001, China; 2 Jiangsu Key Laboratory of Construction Materials, School of Materials Science and Engineering, Southeast University, Nanjing 211189, China

**Keywords:** sodium carbonate, alkali-activated slag, calcined recycled concrete fines, hydration kinetics, compressive strength

## Abstract

Sodium carbonate (Na_2_CO_3_), an environmentally friendly activator, has been shown to have vast potential for the development of sustainable alkali-activated slag mortars. However, Na_2_CO_3_-activated slag mortars exhibit a delayed reaction process and limited early-age strength development, restricting their wider application. In this work, the recycled concrete fines were calcined at a temperature of 800 °C for 1 h and then used as an auxiliary activator to improve the reaction kinetics of Na_2_CO_3_-activated slag mortars. The impact of the calcined recycled concrete fines (CRCF) dosage and Na_2_CO_3_ concentration on the compressive strength, hydration kinetics, and phase assemblage of mortars was evaluated. The results show that CRCF can react directly with Na_2_CO_3_ in the early stages, swiftly removing the CO_3_^2−^ in aqueous solution and providing an alkaline environment suitable for the dissolution of slag. This promotes the development of C-(A)-S-H, hydrotalcite, hemicarbonate, and monocarbonate. The hydration process and strength-giving phase of mortars can be improved further, as an increase in Na_2_CO_3_ concentration increases the initial alkaline content. Additionally, the most remarkable compressive strength value of 39.2 MPa was observed at 28 days in the mortar with 6% sodium oxide equivalent (Na_2_O-E) of Na_2_CO_3_ and 15% CRCF because of the synergistic effect of CRCF and Na_2_CO_3_.

## 1. Introduction

Ordinary Portland cement (OPC) is the most extensively applied cementitious material for concrete production. The high CO_2_ emission and consumption of raw materials, however, are created by OPC production, which results in serious environmental problems such as rising sea levels and global warming [1,2]. To relieve the increasing pressure in OPC production and thus reduce CO_2_ emissions, the alkali-activated slag (AAS) binders produced by the reaction between alkaline activators and granulated blast furnace slag (GBFS) have been rapidly developed in the last few decades. It is reported that a reduction of 55–75% in CO_2_ emissions can be achieved in AAS concrete compared with the traditional cement concretes [3,4]. The cementitious properties of GBFS are majorly determined by the type and concentration of activators due to GBFS dissolution, depending on the pH values of the pore solution [5]. With the development of AAS cements, many activators have been reported in previous studies, with sodium hydroxide (NaOH) and sodium silicate (Na_2_O·nSiO_2_) being the most extensively applied activators, which can ensure that the GBFS is activated to a high degree [4,6,7]. However, the AAS mortars with activators of NaOH and Na_2_O·nSiO_2_ tend to have a high cost, a rapid setting, and micro-cracking issues [8,9]. In addition, a considerable carbon footprint is released in the manufacturing process of NaOH and Na_2_O·nSiO_2_ [6], and, therefore, these activators contribute less to the sustainability of AAS cements.

Some investigations have recently attempted to substitute these strong base activators with sodium carbonate (Na_2_CO_3_). This has been the most popular and promising way to develop sustainable AAS mortars, as the Na_2_CO_3_ can not only easily be obtained from trona ore but is also 2–3 times cheaper than the Na_2_O·nSiO_2_ as well as NaOH [10]. In addition, Na_2_CO_3_, as a near-neutral salt, is less harmful to the handler than strong base activators and releases a lower carbon footprint [1,11]. However, compared to the NaOH and Na_2_O·nSiO_2_, Na_2_CO_3_ provides a relatively weak alkaline environment for the dissolution of GBFS, and, therefore, a slow hydration process with a very long induction period and low early-age strength development can be observed in one-part Na_2_CO_3_-activated GBFS binders [12,13,14]. They even fail to harden after a curing time of more than 28 days at ambient temperature [4,15]. This is an obvious challenge limiting the application of Na_2_CO_3_-activated GBFS.

Recent studies focused on accelerating the reaction kinetics of Na_2_CO_3_-activated GBFS binders by adding some auxiliary activators to increase the OH^−^ in aqueous solution. For example, Dung et al. [16] investigated the influence of magnesium oxide (MgO) on the hydration process of Na_2_CO_3_-activated GBFS binders, and the results indicate that the CO_3_^2−^ in aqueous solution could be rapidly removed by forming hydrotalcite-like phases, which promoted the hydration of GBFS and created more strength-giving phases. Similar results were observed by Fei and Abir [17]. Jeon et al. [18] indicated that calcium hydroxide (Ca(OH)_2_) could effectively promote the hydration of GBFS and improve the early-age compressive strength of mortars activated by Na_2_CO_3_ due to the additional OH^−^ that was introduced. These auxiliary activators exhibit excellent improvements in the acceleration of GBFS hydration, especially at early ages. However, the levels of energy consumption and CO_2_ emission in the preparation process are still high. Therefore, it is necessary to explore an environmentally friendly auxiliary activator.

Recycled concrete fines (RCF) are a by-product from the production of recycled concrete aggregates from waste concrete, and they account for approximately 20–30% of the weight of waste concrete [19,20]. Previous studies demonstrated that the RCF exhibited potential reactivity, which can be used for new concrete production [21]. However, the workability, strength, and durability of concrete containing RCF would significantly decrease due to the high porosity and low reactivity of RCF [22,23,24]. Therefore, only a minimal amount of RCF can be utilized, and the rest is disposed of by landfilling and dumping, failing to take full advantage of waste resources and causing environmental and groundwater pollution [2,19]. It is noteworthy that the major components of RCF, including C-S-H gels, calcite (CaCO_3_), quartz (SiO_2_), Ca(OH)_2_ [20,25], and the high content of CaO, can be obtained in the RCF after thermal treatment (800 °C) due to the decomposition of C-S-H, CaCO_3_, and Ca(OH)_2_. This provides a possibility for calcined recycled concrete fines (CRCF) to become recycled calcium resources or green alkaline supplements in the OPC and AAS binders [26,27]. Indeed, some studies have demonstrated that the CaO could accelerate the reaction of Na_2_CO_3_-activated GBFS binders and improve their early-age strength [7,28]. Wang et al. [4] observed that the reaction kinetics of Na_2_CO_3_-activated GBFS could be accelerated by calcium oxide (CaO), and the mortars with 5% Na_2_CO_3_ and 2.5% CaO by mass of GBFS exhibited a high early-age strength due to the removal of CO_3_^2−^.

In this case, CRCF should be developed as an auxiliary activator for accelerating the reaction kinetics in the Na_2_CO_3_-activated slag binder. This can alleviate the long-term piling problem of RCF. Additionally, this can decrease the energy consumption and CO_2_ emissions generated by traditional auxiliary activators (generally calcined at more than 1000 °C). However, few studies have investigated this area, and it is therefore necessary to fill the research gaps from previous studies. There is no doubt that the content of CRCF and Na_2_CO_3_ is a main factor that influences the reaction process of blended pastes and mortars. Therefore, AAS mortars and pastes with different amounts of Na_2_CO_3_ and CRCF were prepared in this work. Then, we tested the strength of blended mortars, which was performed to assess the influence of CRCF and Na_2_CO_3_ on the mortar mechanical performance. A heat evolution test was employed to study the hydration process of the blended pastes during the first 120 h. In addition, X-ray diffraction (XRD), mercury intrusion porosimetry (MIP), Fourier transform infrared spectrometry (FTIR), and thermogravimetric analysis (TGA) were performed to assess the reaction products and microstructures of the mortars.

## 2. Materials and Methods

### 2.1. Materials

**Figure 1:**
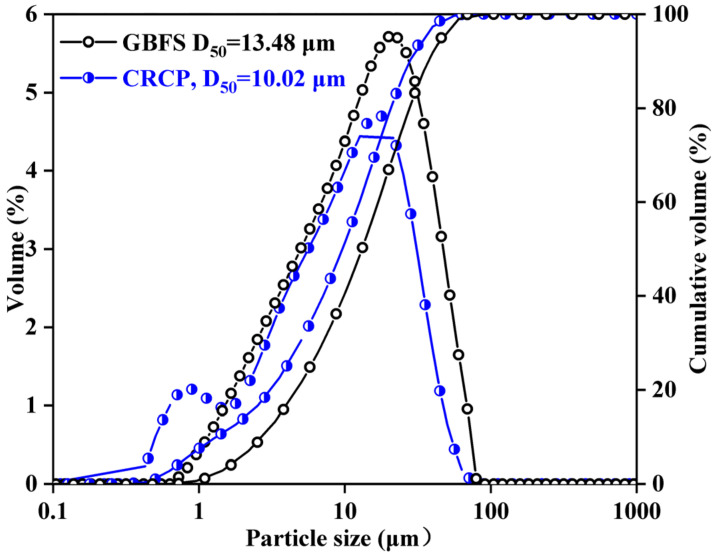
Particle size distribution of GBFS and CRCF.

Granulated blast furnace slag (GBFS) and calcined recycled concrete fines (CRCF) were used as binders in this study. The chemical compositions were measured by an XRF-1800 sequential X-ray fluorescence instrument, and the results are listed in Table 1. The Al_2_O_3_ and MgO content in GBFS was much higher than that in CRCF, while the opposite was found for the CaO content. Figure 1 shows the particle size distribution of GBFS and CRCF. The median diameter (D_50_) was used to characterize their fineness, and the D_50_ of GBFS and CRCF was 13.48 and 10.02, respectively, demonstrating that the CRCF was thicker than the GBFS. The sodium carbonate (Na_2_CO_3_) powder, which was purchased from Tianjin Hengxing Chemical Reagent Manufacturing Co., LTD. (Tianjin, China), was used as an activator and marked as sodium oxide equivalent (Na_2_O-E). River sand with a fineness modulus of 2.56 and an apparent density of 2550 kg/m^3^ was used as the fine aggregate. City tap water was used to dissolve the Na_2_CO_3_ powder and prepare the activator solution.

### 2.2. Preparation and Characterization of CRCF

In this work, the recycled concrete fines (RCF) were prepared by grinding the laboratory-made cement mortar specimens with a water/cement ratio of 0.5 and a sand/cement ratio of 1. These mortar specimens were cured in a curing room with an ambient temperature of 23 ± 2 °C and a relative humidity of no less than 95% for 28 days, and they were left in the air for three months. The real RCF, normally prepared from recycled concrete aggregates, has a more complex composition, including crushed rock minerals and other supplementary cementitious materials (e.g., fly ash). The use of pure cement mortars in the study provided a starting point for further research. The CRCF was produced by calcining the RCF in a Muffle furnace with a temperature of 800 °C and a holding time of 1 h. Then, this was stored in the furnace and cooled to room temperature. Once the cooling process was finished, the samples of CRCF were sealed and stored in plastic bags until further tests. The time and temperature of this thermal treatment procedure were obtained from previous studies [26].

**Figure 2:**
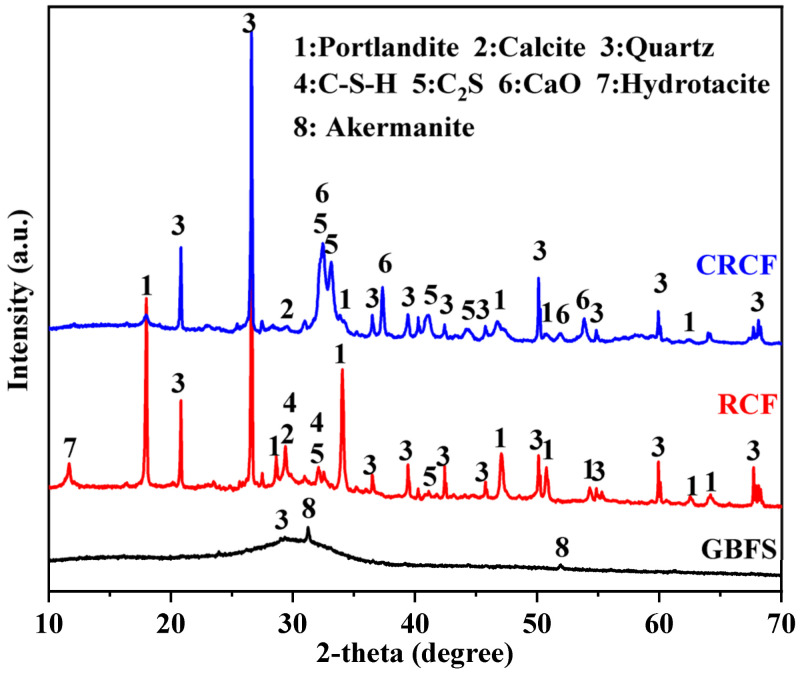
XRD patterns of GBFS, CRCF, and RCF. Phases identified: Portlandite PDF No. 44–1481; Calcite PDF No. 05–0586; Quartz PDF No. 46–1045; C-S-H PDF No. 33–0306; C_2_S PDF No. 29–0369; CaO PDF No. 28–0775; Hydrotalcite PDF No. 89–0460; and Akermanite PDF No. 76–0841.

Figure 2 shows the mineralogical phases of GBFS, RCF, and CRCF. The GBFS showed an amorphous phase in the range of 2θ = 10–70° with some characteristic peaks of akermanite (2θ = 31.3° and 2θ = 51.9°) and calcite (2θ = 29.4°). Portlandite, calcite, and quartz were the major crystalline phases of RCF. Additionally, a peak of unhydrated C_2_S was observed in XRD patterns. After thermal treatment, the portlandite, calcite, and C-S-H phases almost disappeared. The peaks representing CaO were identified and were mainly attributed to the decarbonization of calcite and the dehydration of portlandite and C-S-H. During thermal treatment, the high-activity CaO can react with SiO_2_ to form the incomplete crystallization of C_2_S [29]. In addition, the C_2_S can also be formed by the condensation polymerization of Ca-O-Si bonds in the amorphous macromolecular groups produced by the early dehydration of C-S-H [30]. Therefore, the incomplete crystallization of the C_2_S phase was observed in CRCF and had the ability to form gel products.

### 2.3. Mortar Specimen Preparation

To assess the influence of CRCF dosage and Na_2_CO_3_ concentration on the reaction kinetics of binder systems, we prepared two series of mortar specimens in accordance with Table 2. The first series was manufactured as follows: the Na_2_O-E was fixed at 5%, and the dosage of CRCF was 0, 5, 10, 15, 20, and 30% by weight of binders, respectively. In the second series, the amount of CRCF was kept at 15%, while the Na_2_O-E was increased from 3 to 7% with a spacing of 1%. For all the mortar specimens, the water-to-binder-mass ratio was fixed at 0.45, and the binder-to-sand ratio was kept at 0.69. Table 2 provides the details of the mixed proportions of mortars.

All of the mixtures were prepared in the same steps. The Na_2_CO_3_ powder was first dissolved in water and cooled to room temperature. Then, the sand, GBFS, and CRCF were homogenized in a mechanical mixing pot for 2 min. Afterward, the Na_2_CO_3_ solution was added and stirred for another 2 min. The fresh mortars were cast into cubic molds with a size of 50 mm^3^ and vibrated on a vibration table with a time of 30 s. After this, the fresh mortar specimens were cured in a curing room with a temperature of 23 ± 2 °C and a minimum relative humidity of 95%. After the mortars hardened, they were demolded and continued to cure for 3, 7, and 28 days. In addition, the selected paste specimens, which were named CR0S5, CR5S5, CR15S5, CR30S5, CR15S3, and CR15S7, were prepared according to Table 2 (without sand) for the identification of hydration products, and they were cured in the same conditions with mortars for 3 days and 28 days.

### 2.4. Test Methods

#### 2.4.1. Hydration Heat

The hydration heat flow evolution of CR0S5, CR15S5, CR30S5, CR15S3, and CR15S7 pastes was determined by using a TAM-AIR isothermal calorimeter to evaluate the effect of CRCF dosage and Na_2_CO_3_ concentration on the reaction kinetics of binder systems in accordance with ASTM C1702-17 [31]. The heat released from blended pastes was recorded automatically for the first 120 h.

#### 2.4.2. Hydration Products

The paste specimens were first crushed into small pieces, which were soaked in anhydrous ethanol to prevent further hydration for 24 h. Then, these small pieces were dried in a freeze-drying oven for 24 h and ground into a fine powder with a particle size no greater than 75 μm.

The mineralogical phases of these fine powder samples were identified by a Smart lab SE type X-ray diffraction (XRD) instrument (Columbus, OH, USA). The scanning speed and test range for each sample were 5°/min and 10–40°, respectively.

The phase compositions of paste samples were determined by using a Simultaneous Thermal Analyzer 8000 (Perkin Elmer, Waltham, MA, USA) under an N_2_ atmosphere. The sample mass was recorded automatically from 30 °C to 800 °C at 20 °C/min.

Fourier transform infrared spectrometry (FTIR) was carried out using a Thermo Scientific Nicolet IS50 (Waltham, MA, USA) to identify the chemical bonds of the paste samples with a frequency range between 4000 cm^−1^ and 400 cm^−1^.

#### 2.4.3. Pore Structure

The samples (approximately 1 cm^3^) were first collected from the central portions of the mortar specimens and soaked in anhydrous ethanol to prevent further hydration for 24 h. After this, they were dried in a freeze-drying oven for 24 h. Subsequently, the pore structure evolution of these samples was measured by an AutoPoreIv 9510 MIP instrument.

#### 2.4.4. Compressive Strength

The compressive strength testing of mortar specimens was carried out according to the ASTM C109 [32] standard. Three samples were measured in each group using a compression testing machine, and the final results were obtained by calculating their average value.

## 3. Results and Discussion

### 3.1. Hydration Heat

**Figure 3:**
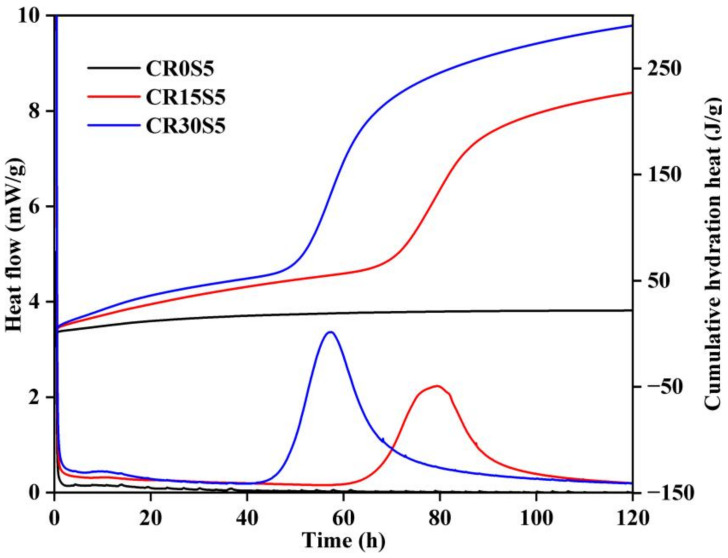
Hydration heat of pastes with different CRCF dosages.

Figure 3 displays the heat release of blended pastes with different amounts of CRCF. There was an initial reaction during the period of 0–0.5 h, involving the chemical reaction between Na_2_CO_3_ and CRCF and the initial dissolution and wetting of the binders [2]. Ultimately, due to extended stirring, the heat release was not captured in the initial period. After this stage, a long-term induction period began and exhibited low heat evolution, which is associated with the formation of the initial reaction products (e.g., C-(A)-S-H, calcite, and gaylussite); this was also observed in previous studies [33,34]. It can be found that the induction period was shortened by the addition of CRCF. This indicated that incorporating CRCF accelerated the reactivity of mixtures due to the additional OH^−^ generated by the reaction between Na_2_CO_3_ and CRCF. Second peaks then appeared, with times of 45–70 h and 65–100 h in the pastes CR15S5 and CR30S5, respectively, which related to the acceleration–deceleration process pertaining to the precipitation of hydration products, especially the polymerization of C-(A)-S-H. However, no second exothermic peak was observed in paste CR0S5 during the first 120 h. This indicated that a slow reaction occurred in the one-part Na_2_CO_3_-activated GBFS system. A similar result was reported by Gao et al. [12], who observed an induction period over 72 h of Na_2_CO_3_-activated GBFS with 8 wt.% of Na_2_CO_3_ by mass of GBFS and a water-to-binder ratio of 0.53. When the CRCF was added, the CO_3_^2−^ of the reaction system was consumed by CRCF, which increased the amounts of OH^−^ and accelerated the polymerization of C-(A)-S-H gels. In addition, more reactive Ca was released from the CRCF, which promoted the formation of hydration products (e.g., C-(A)-S-H, hydrotalcite, and hemicarbonate). Therefore, the second peak can be observed in pastes with CRCF. Compared to paste CR15S5, the higher second peak intensity and earlier peak time were identified in paste CR30S5. Pastes CR15S5 and CR30S5 reached their second peaks at approximately 79.5 and 57.3 h, respectively, and their peak intensities were 2.2 and 3.4 mW/g. This was a result of the greater availability of OH^−^ and Ca^2+^ produced by a high CRCF dosage, which provided a decent alkali environment for forming hydration products. Due to the acceleration of CRCF on the Na_2_CO_3_-activated GBFS system, the cumulative hydration heat of pastes with CRCF was increased from 0 to 30% with increasing CRCF replacement levels.

**Figure 4:**
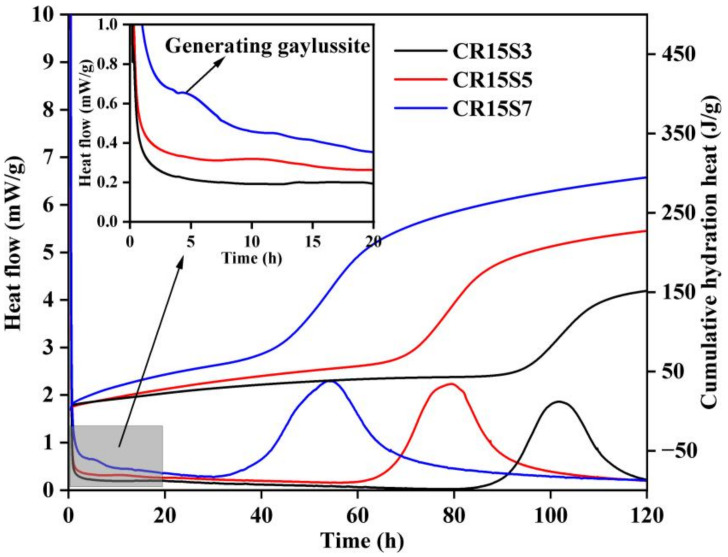
Hydration heat of pastes with different Na_2_CO_3_ concentrations.

The influence of Na_2_CO_3_ concentration on the heat evolution process of blended pastes is given in Figure 4. It can be seen that the hydration of binders was accelerated with increasing Na_2_CO_3_ content. For example, the second peak intensity was 1.9, 2.2, and 2.3 mW/g for CR15S3, CR15S5, and CR15S7, respectively. In addition, both the induction period and the second peak time were shortened by the increase in Na_2_CO_3_ concentration. These phenomena could be attributed to the high degree of reaction between the activator and CRCF. In the aqueous solution, the CRCF directly reacted with Na_2_CO_3_ and generated CaCO_3_, which resulted in the rapid consumption of CO_3_^2−^ while also increasing the amount of OH^−^, which determined the dissolution degree of GBFS [6]. When the binders were activated by a low concentration (Na_2_O-E: 3%) of Na_2_CO_3_, insufficient CO_3_^2−^ reacted with Ca^2+^ released from CRCF, leading to a relatively low level of OH^−^ in the aqueous solution. This limited the dissolution of GBFS and decreased the polymerization degree of C-(A)-S-H gels. High amounts of Na_2_CO_3_ not only increased the initial alkali content but also accelerated the reaction between Na_2_CO_3_ and CRCF and generated more amounts of OH^−^, which further promoted the dissolution of GBFS. Therefore, the hydration heat evolution was enhanced by the increasing Na_2_CO_3_ concentration. It should be noted that a weak peak was detected in paste CR15S7 at approximately 4 h, corresponding to the formation of gaylussite, which was caused by superfluous CO_3_^2−^. This was also found in the previous literature [12].

### 3.2. XRD Analysis

**Figure 5:**
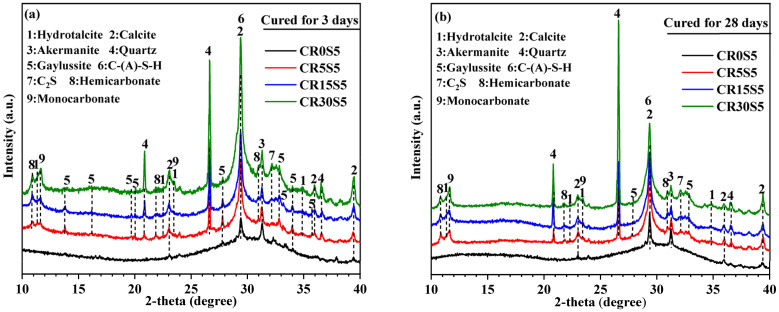
XRD patterns of paste samples with different CRCF dosages: (**a**) cured for 3 days and (**b**) cured for 28 days. Phases identified: Hydrotalcite PDF No. 89–0460; Calcite PDF No. 05–0586; Akermanite PDF No. 76–0841; Quartz PDF No. 46–1045; Gaylussite PDF No. 74–1235; C-(A)-S-H PDF No. 89–6548; C_2_S PDF No. 29–0369; Hemicarbonate PDF No. 41–0221; and Monocarbonate PDF No. 41–0219.

Figure 5 shows the XRD patterns of paste samples with different CRCF dosages at 3 days and 28 days, respectively. Akermanite, as the major crystalline phase of GBFS, was detected in all the samples regardless of the amount of CRCF and the curing time, which was in good agreement with previous studies [4,35].

From Figure 5a, the major crystalline phases in paste samples with CRCF were hydrotalcite (Mg_6_Al_2_CO_3_(OH)_16_·4H_2_O), calcite, gaylussite (Na_2_Ca(CO_3_)_2_·5H_2_O), C-(A)-S-H, hemicarbonate (Ca_4_Al_2_(OH)_13_(CO_3_)_0.5_(H_2_O)_5_), and monocarbonate (Ca_4_Al_2_(OH)_12_(CO_3_) (H_2_O)_5_), along with quartz, C_2_S, and calcite from the CRCF. However, only some weak diffraction peaks of calcite, gaylussite, and C-(A)-S-H were identified in the XRD patterns of pure slag paste, except for akermanite, due to the slow hydration of GBFS. Calcite and gaylussite were the major carbonates in all the paste samples with CRCF, which were influenced by the amounts of CRCF. For example, the diffraction peak intensities of the gaylussite phase were decreased with increasing replacement levels of CRCF, and some peaks even disappeared in sample CR30S5. The opposite phenomenon was observed in the diffraction peak intensities of the calcite phase. This is attributed to the reaction between CRCF and Na_2_CO_3_, which consumed CO_3_^2^^−^ and increased the amounts of CaCO_3_ and OH^−^. There was no doubt that more CaCO_3_ could be generated when more CRCF was included. Meanwhile, the reduction of CO_3_^2−^ in the pore solution contributed to the decomposition of gaylussite and further formed CaCO_3_.

For the other carbonates such as hydrotalcite (Ht), hemicarbonate (Hc), and monocarbonate (Mc), their diffraction peak intensities increased with increasing CRCF dosages. This indicated that the CRCF could promote the formation of carbonates, except for the gaylussite. In addition, the diffraction peaks of the gaylussite phase in all samples at 28 days (Figure 5b) almost disappeared, regardless of the amounts of CRCF, which was related to the production of more stable carbonates [35]. We discuss this in detail in Section 3.7.

**Figure 6:**
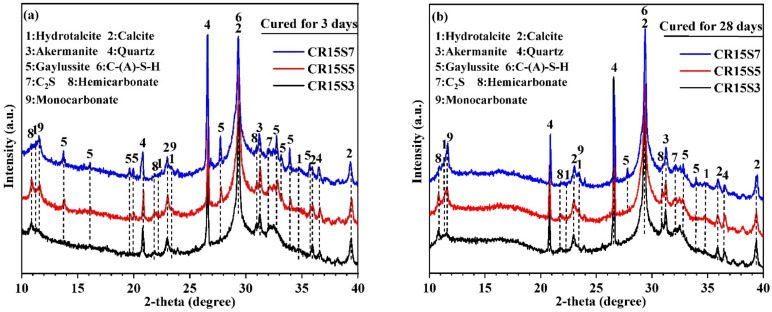
XRD patterns of paste samples with different Na_2_CO_3_ concentrations: (**a**) cured for 3 days and (**b**) cured for 28 days. Phases identified: Hydrotalcite PDF No. 89–0460; Calcite PDF No. 05–0586; Akermanite PDF No. 76–0841; Quartz PDF No. 46–1045; Gaylussite PDF No. 74–1235; C-(A)-S-H PDF No. 89–6458; C_2_S PDF No. 29–0369; Hemicarbonate PDF No. 41–0221; and Monocarbonate PDF No. 41–0219.

The influence of Na_2_CO_3_ concentration on the hydration products of paste samples with 15% CRCF at 3 days and 28 days is displayed in Figure 6. For the samples after 3 days of curing (Figure 6a), they exhibited the same crystalline phases as the samples with different CRCF dosages (Figure 5a). As the Na_2_CO_3_ concentration increased from 3% to 7%, the diffraction peak intensities of the gaylussite, Ht, and Mc phases gradually increased. This was due to the availability of CO_3_^2−^ presented in the pore solution, which provided suitable conditions for its reaction with Ca^2+^ and Na^+^, Mg^2+^, and [Al(OH)_4_]^−^ to generate more carbonates (gaylussite, Ht, and Mc). In fact, the Mc was more thermodynamically stable compared with the Hc [36], and the Hc can be converted to Mc under a high CO_3_^2−^ concentration. Additionally, this was a reason for the peak intensities of Mc to have increased with the increase in Na_2_CO_3_ concentration. For the XRD patterns of the samples at 28 days (Figure 6b), the diffraction peak intensities of Hc were further decreased. This indicated that a high Na_2_CO_3_ concentration can not only promote the conversion from Hc to Mc but also increase the curing time. In addition, some peaks of gaylussite disappeared because it was a transient phase.

### 3.3. FTIR Analysis

**Figure 7:**
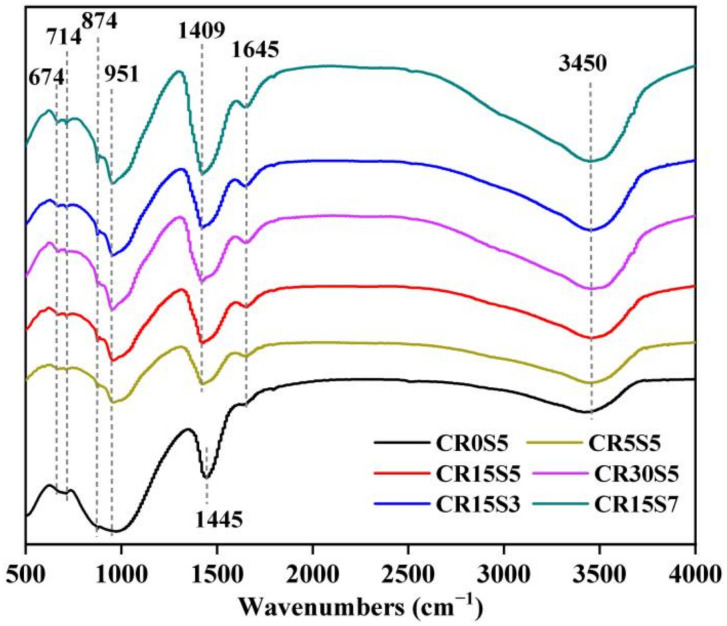
FTIR spectrum of paste samples at 28 days.

Figure 7 shows the FTIR spectrum of selected paste samples at 28 days. The traced absorption peaks at 674 cm^−1^ can be attributed to the vibration of Al-O bands in the AlO_4_ groups, which resulted from the incomplete hydration of GBFS [37]. The absorption peaks of 714 cm^−1^ and 874 cm^−1^ were the result of the vibration of *v*_2_[CO_3_^2−^] and *v*_4_[CO_3_^2−^], respectively, and the absorption peaks around 1409 cm^−1^ and 1445 cm^−1^ can be attributed to the vibration of *v*_3_[CO_3_^2−^] [38]. For the sample CR0S5, the CO_3_^2–^ was predominantly derived from the calcite, while it was derived from carbonates such as glayssuite, Ht, Hc, Mc, and calcite for other samples according to the XRD results. The peak traced at 1445 cm^−1^ shifted to the low-wavelength range (1409 cm^−1^) with the addition of CRCF regardless of the Na_2_CO_3_ concentration, which could potentially be attributed to the formation of gaylussite, consistent with a previous study [37]. The absorption peaks located at 1645 cm^−1^ and 3450 cm^−1^ were generally regarded as the -OH bending vibration mode in chemically bound water and as the H-O-H stretching vibration mode in free water, respectively. The absorption bands at 951 cm^−1^ represented typical asymmetric Si-O stretching vibration related to the C-(A)-S-H gels [39]. The peak intensity was increased with the addition of CRCF and was further improved by a higher concentration of Na_2_CO_3_. This indicated that the incorporation of CRCF promoted the formation of C-(A)-S-H gels and was further enhanced by increasing the concentration of Na_2_CO_3_.

### 3.4. TG-DTG Analysis

**Figure 8:**
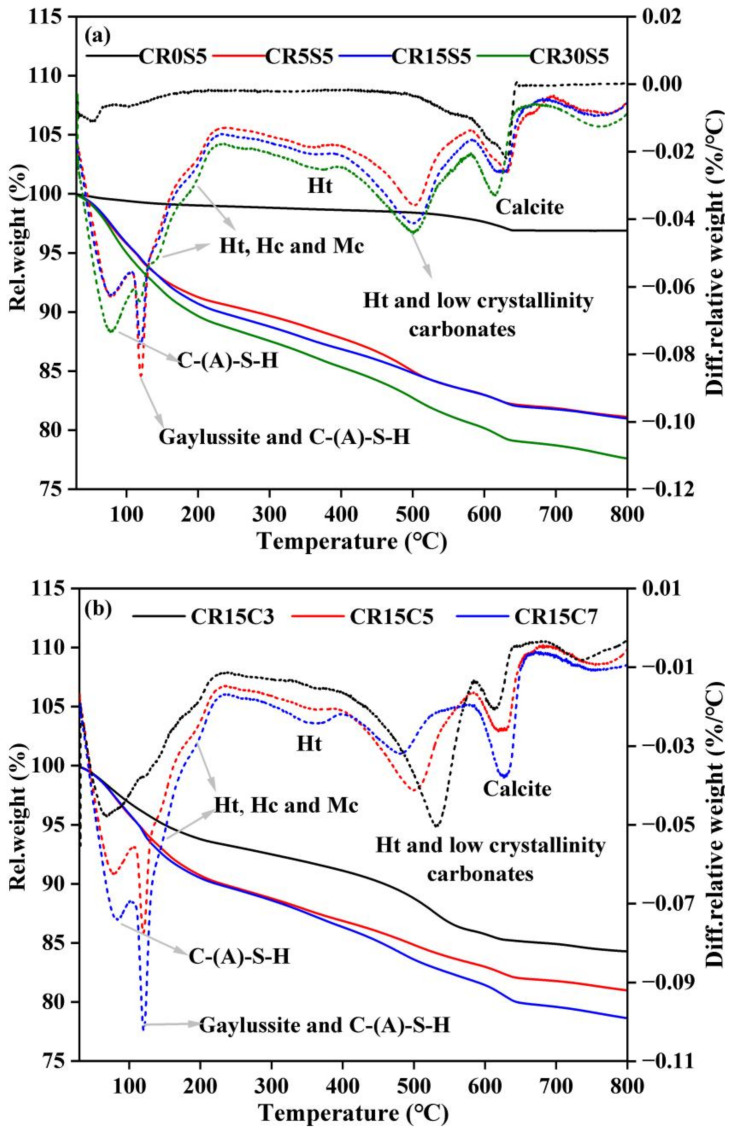
TG-DTG curves for the paste samples at 3 days: (**a**) different CRCF dosages and (**b**) different Na_2_CO_3_ concentrations.

The TG-DTG curves of paste samples with different CRCF dosages and Na_2_CO_3_ concentrations after 3 days are given in Figure 8. From Figure 8a, four major peaks can be observed in the DTG curves of paste samples with CRCF, corresponding to the four areas of rapid weight loss in the TG curves. The first peak was 40–250 °C due to the dehydration of C-(A)-S-H gels and carbonates such as Ht, Hc, and Mc, as identified by XRD and FT-IR [9]. Of note, the peak located at approximately 120 °C was mainly attributed to the dehydration of gaylussite, and its intensity decreased with an increasing CRCF dosage. This was in agreement with the XRD results in Section 3.2. The second peak between 300 and 400 °C resulted from the dehydroxylation of Ht and has been reported by many researchers [4]. The peaks within the temperature ranges of 450–600 °C and 600–700 °C were caused by the decarbonization of Ht and low-crystallinity carbonates and the decarbonation of calcite, respectively [39]. For sample CR0S5, only two peaks could be detected, in the ranges of 40–250 °C and 600–700 °C, which is related to the dehydration of C-(A)-S-H and the decarbonation of calcite. This is in agreement with the results from the XRD patterns (Figure 5a). There were no newly formed peaks in the DTG curves of the paste samples with different amounts of Na_2_CO_3_ (Figure 8b) compared with the peaks in the samples with different CRCF dosages, except for the sample CR0S5, suggesting that the concentration of Na_2_CO_3_ cannot affect the type of hydration products.

Although each hydration product has its own decomposition temperature range, it is still difficult to distinguish between them due to the overlaps in some temperature ranges. For example, the peak between 40 °C and 150 °C was attributed to the dehydration of C-(A)-S-H based on a previous study [40]. However, the peak located at approximately 120 °C was mainly attributed to the dehydration of gaylussite, affecting the accurate amount of C-(A)-S-H phase by TGA quantification. Therefore, the mass loss of paste samples at different temperature ranges was calculated as an evaluation indicator for the degree of reaction, as summarized in Table 3. The mass loss of each temperature range in sample CR0S5 was lower than that in other samples, whether 3 days or 28 days, which indicated that it had a low degree of reaction. When the CRCF was added, the mass loss of each temperature range significantly increased, and more improvement can be achieved by the further addition of CRCF. This is due to the additional OH^−^ produced by the reaction between CRCF and Na_2_CO_3_, which accelerated the hydration of GBFS and induced the conversion of gaylussite to other carbonates. For the paste samples with different amounts of Na_2_CO_3_, the mass loss between 450 and 600 °C was decreased with the increase in Na_2_CO_3_ concentration, while the mass loss in the range of 600–700 °C increased, which indicated that a high concentration of CO_3_^2−^ can induce the conversion of low-crystallinity carbonates to high-crystallinity carbonates such as calcite. In addition, the mass loss with temperatures of 40–250 °C and 300–400 °C was increased with an increasing Na_2_CO_3_ concentration, which confirmed that a high concentration of Na_2_CO_3_ could promote the reaction of GBFS. It should be noted that the increased mass loss of all samples from 3 days to 28 days demonstrates the reaction process and the formation of hydration products.

### 3.5. Pore Structure Evolution

**Figure 9:**
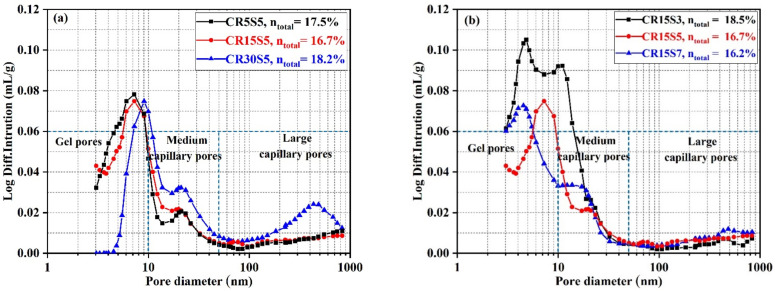
The pore structure distribution curves of blended mortars at 28 days: (**a**) the influence of CRCF dosage and (**b**) the effect of Na_2_O-E concentration.

The pore structure evolution of blended mortars is shown in Figure 9. From Figure 9a, it can be seen that the pores of all samples were majorly distributed within 3–1000 nm. According to a previous report, the pores in alkali-activated cementitious materials can be divided into gel pores, medium-capillary pores, and large-capillary pores, corresponding to pore sizes of less than 10 nm, 10–50 nm, and 50–1000 nm, respectively [12]. The number of gel pores decreased with the increase in CRCF, and the opposite phenomenon was observed in medium-capillary pores. This indicated that a high CRCF dosage limited the conversion of medium-capillary pores to gel pores. In other words, C-(A)-S-H, a major hydration product filling the pores, was decreased by an increasing CRCF dosage. It should be noted that a weak peak was observed at approximately 432.7 nm in the CR30S15 mortar, which was due to the excess CRCF significantly decreasing the amounts of C-(A)-S-H gels and the gaps in the mortar not being filled effectively. This is also the reason why the CR30S15 exhibited the highest total porosity (n_total_). Although the medium-capillary pores in CR15S5 were increased compared with the CR5S5 mortar, the n_total_ in the CR15S5 mortar was lower than that in the CR5S5 mortar due to the filler effect of CRCF. The effect of Na_2_CO_3_ concentration on the pore structure evolution of mortars is displayed in Figure 9b. An obvious improvement in the pore structure of mortars can be clearly seen with an increase in the concentration of Na_2_CO_3_, and the n_total_ is 18.5, 16.7, and 16.2% for the CR15S3, CR15S5, and CR15S7 mortars. This can be attributed to the initial alkaline concentration being enhanced by the growth of Na_2_CO_3_ dosage, which promoted the dissolution of GBFS and accelerated the formation of C-(A)-S-H to refine the pores. It is noteworthy that the number of medium-capillary pores in the CR15S5 mortar is smaller than that in the CR15S7 mortar due to more calcite being formed in the CR15S5 mortar, which created a relatively coarser pore structure [41].

### 3.6. Compressive Strength

**Figure 10:**
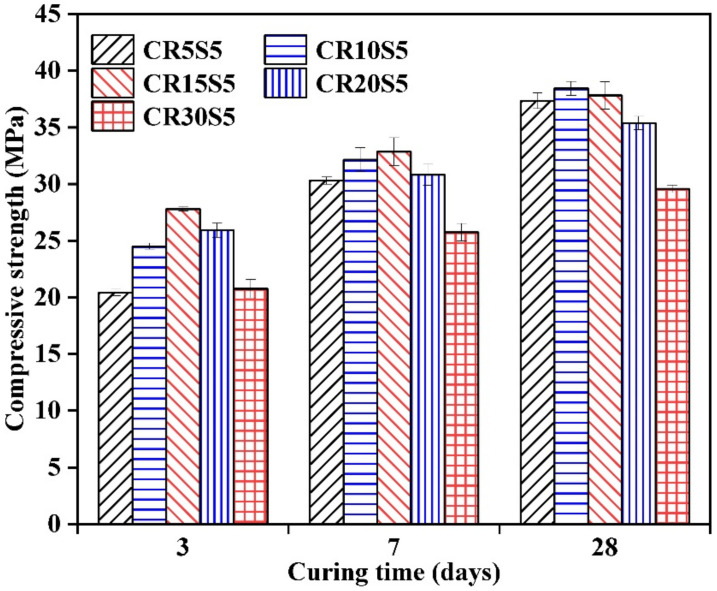
Compressive strength of mortars with different amounts of CRCF.

The influence of CRCF dosage on the compressive strength of mortars is exhibited in Figure 10. There was a failure to harden for the mortars activated by one-part Na_2_CO_3_, and, therefore, no compressive strength was measured. This is consistent with the research of Wang et al., who used Na_2_CO_3_-activated GBFS with 4 wt.% of Na_2_O-E by mass of slag and a water-to-binder ratio of 0.4 and found that the mortar failed to activate [16]. A different result, however, was reported by Zhang et al. [42], who used 3.42–10.26% by weight of slag to activate the GBFS mortars and achieved 23.1–61.3 MPa for the compressive strength of mortars at 28 days. This difference could be attributed to the discrepancies in the physical and chemical properties pertaining to GBFS, and the GBFS used in this work was coarser than that in Zhang et al. [42].

With the addition of CRCF, the hydration of GBFS was accelerated, and the mortars exhibited a high compressive strength at an early age. For example, the strength values of the CR5S5, CR10S5, CR15S5, CR20S5, and CR30S5 mortars were 20.4, 24.5, 27.8, 25.9, and 20.8 MPa at 3 days, respectively, and the CR15S5 obtained the highest compressive strength. Generally, the C-(A)-S-H gels produced by GBFS hydration played a major role in the compressive strength improvement of mortars [40]. In the process of early-age hydration, the OH^−^ was rapidly formed by the reaction between Na_2_CO_3_ and CRCF, which promoted the dissolution of GBFS and increased the strength-giving phase in substances such as C-(A)-S-H gels. In addition, the C_2_S in CRCF can involve the hydration reaction and generate C-S-H gels to enhance the compressive strength of mortars. Due to the replacement of a considerable proportion of GBFS by CRCF (more than 15%), which decreased the amount of C-(A)-S-H gel and increased the total porosity of the mortar, the compressive strength of the mortar was decreased. When the curing time reached 28 days, the highest strength value (38.4 MPa) was observed in the CR10S5 mortar rather than the CR15S5 mortar. This was predominantly due to the incomplete hydration of GBFS at 3 days; meanwhile, more CRCF was included in the mixtures, resulting in a stronger acceleration effect based on the results of hydration heat. With the continuous hydration of GBFS, the high-dosage CRCF led to a decrease in C-(A)-S-H gels due to the reduction in GBFS content.

**Figure 11:**
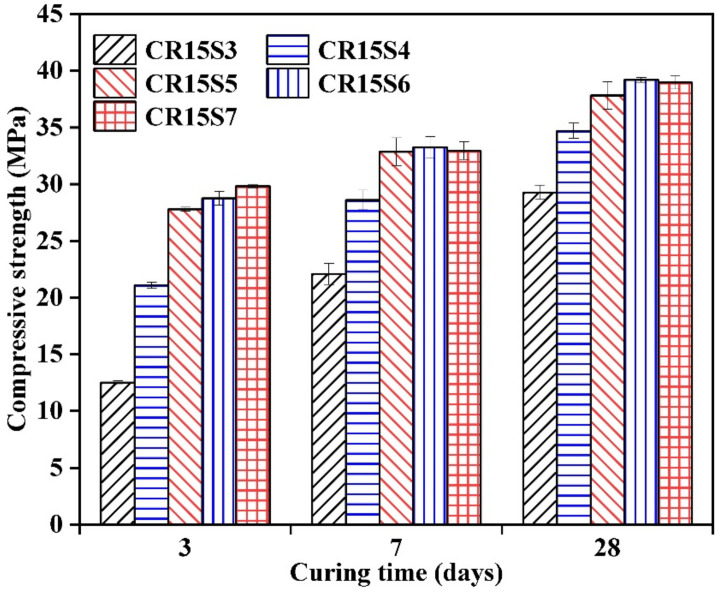
Compressive strength of mortars with different Na_2_CO_3_ contents.

Figure 11 gives the compressive strength of 85% GBFS and 15% CRCF mortars blended with different amounts of Na_2_CO_3_. It can be clearly seen that the compressive strength of mortars was obviously increased by high NaO-E at all ages. Compared to the CR15S3 mortar, the compressive strength was increased by 68.7%, 122.3%, 130.0%, and 138.6% for the CR15S4, CR15S5, CR15S6, and CR15S7 mortars at 3 days, respectively. This indicated that the amounts of Na_2_CO_3_ were a major factor controlling the compressive strength at an early age. When the amounts of NaO-E reached 5% by mass of GBFS, the improvement effect of Na_2_CO_3_ on the compressive strength of mortars was less marked. Even a slight reduction was observed in the compressive strength of the CR15S7 mortar compared with the CR15S6 mortar at 7 days and 28 days. This was due to the excess Na_2_CO_3_ limiting the pore structure development of mortars and decreasing the compressive strength of mortars. Additionally, Yang et al. [41] demonstrated that a high concentration of CO_3_^2−^ promoted the formation of calcite in the blended system and resulted in a relatively coarser pore structure.

### 3.7. Acceleration Mechanism of CRCF on the Na_2_CO_3_-Activated GBFS Mortars

**Figure 12:**
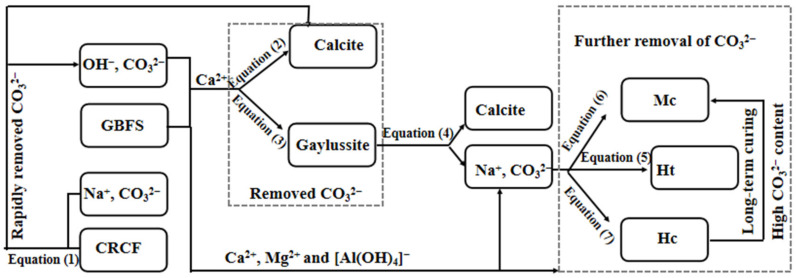
Simplified flow chart of CO_3_^2−^ consumption in mortars with and without CRCF.

For the Na_2_CO_3_-activated GBFS mortars, removing the CO_3_^2−^ in the aqueous solution was the key to promoting GBFS hydration. The conceptual model pertaining to the major consumption process of CO_3_^2−^ in Na_2_CO_3_-activated GBFS mortars with CRCF is exhibited in Figure 12. The Na_2_CO_3_ can react directly with CRCF, swiftly remove the CO_3_^2−^ in an aqueous solution, and produce OH^−^, as shown in Equation (1). This promoted the breakage of Ca-O and Mg-O, as well as Al-O and Si-O, in GBFS and induced the production of the strength-giving phase (C-(A)-S-H). The remaining CO_3_^2−^ would continue to be consumed by forming carbonates such as calcite (Equation (2)), gaylussite (Equation (3) [1]), Ht (Equation (5) [16]), Mc (Equation (6) [43]), and Hc (Equation (7) [43]), as identified in the XRD patterns. It should be noted that the gaylussite, as a transient phase, can be decomposed (Equation (4)) in an environment of low CO_3_^2−^ concentration or high OH^−^ concentration, and the CO_3_^2−^ could be stored in other carbonates (Ht, Hc, and Mc) [44]. However, no peaks of Ht, Hc, and Mc could be detected in one-part Na_2_CO_3_-activated GBFS binders based on XRD patterns (Figure 5) at either 3 or 28 days; meanwhile, the minimal mass loss (%) was obtained in the CR15S5 paste in accordance with the TGA results (Table 3). This indicated that it was difficult to form hydration products in the one-part Na_2_CO_3_-activated GBFS system due to the Na_2_CO_3_ providing a weak alkali environment for the mixed system. This was also exhibited by an induction period of over 120 h in hydration heat evolution (Figure 4) and by the failure of the CR0S5 mortar to harden for more than 28 days (Figure 11).

(1) $${\mathrm{CO}}_{3}{}^{2-}{+\mathrm{CaO}+H}_{2}O\;\to {\;\mathrm{CaCO}}_{3}↓{+2\mathrm{OH}}^{-}$$

(2) $${\mathrm{Ca}}^{2+}{+\mathrm{CO}}_{3}{}^{2-}\to {\;\mathrm{CaCO}}_{3}↓$$

(3) $${5H}_{2}{O+2\mathrm{CO}}_{3}{}^{2-}{+\mathrm{Ca}}^{2+}{+2\mathrm{Na}}^{+}\;\to {\;\mathrm{Na}}_{2}{\mathrm{Ca}(\mathrm{CO}}_{3}{)}_{2}\cdot {5H}_{2}O↓\;(\mathrm{gaylussite})\;$$

(4) $${\mathrm{Na}}_{2}{\mathrm{Ca}(\mathrm{CO}}_{3}{)}_{2}\cdot {5H}_{2}O\;\to {\;5H}_{2}{O+\mathrm{CaCO}}_{3}↓{+2\mathrm{Na}}^{+}{+\mathrm{CO}}_{3}{}^{2-}$$

(5) $$H_{2}{O+\mathrm{OH}}^{-}+[\mathrm{Al}{\left(\mathrm{OH}\right)}_{4}{]}^{-}{+\mathrm{Mg}}^{2+}{+\mathrm{CO}}_{3}{}^{2-}\to \;\mathrm{hydrotalcite}↓$$

(6) $$H_{2}{O+\mathrm{OH}}^{-}+[\mathrm{Al}{\left(\mathrm{OH}\right)}_{4}{]}^{-}{+\mathrm{Ca}}^{2+}{+\mathrm{CO}}_{3}{}^{2-}\to \;\mathrm{hemicarbonate}↓$$

(7) $$H_{2}{O+\mathrm{OH}}^{-}+[\mathrm{Al}{\left(\mathrm{OH}\right)}_{4}{]}^{-}{+\mathrm{Ca}}^{2+}{+\mathrm{CO}}_{3}{}^{2-}\to \;\mathrm{monocarbonate}↓$$

From what has been discussed above, the Na_2_CO_3_, as a major activator of GBFS, exhibited a slow reaction process. The CRCF can work as a reaction source of sodium carbonate to consume the CO_3_^2−^ and accelerate the hydration kinetics of one-part Na_2_CO_3_-activared GBFS binders. Although this acceleration can be improved by a high CRCF dosage, which is observed in the hydration heat results, it exhibited a negative effect on the long-age compressive strength of mortars. The MIP results also demonstrate that the n_total_ of mortars increased with increasing CRCF dosages due to the low activity of CRCF. Therefore, CRCF, as an auxiliary activator, played the main role in controlling the early-age reaction kinetics of Na_2_CO_3_-activared GBFS mortars, rather than enhancing the overall degree of reaction.

## 4. Conclusions

In this work, we attempt to accelerate the reaction kinetics of one-part Na_2_CO_3_-activated GBFS mortars by the addition of CRCF. The effect of CRCF dosage and Na_2_CO_3_ concentration on the compressive strength, hydration kinetics, and phase assemblage of mortars was evaluated. The concluding remarks that follow were drawn in accordance with the results obtained in this study.

(1) The GBFS was activated by one-part Na_2_CO_3_ and exhibited a slow reaction process with a long induction period (more than 120 h). CRCF incorporation significantly accelerated the dissolution of GBFS. In addition, the reaction kinetics can be further improved by increasing the Na_2_CO_3_ content.
(2) The major phase assemblages of AAS mortars with different amounts of CRCF and Na_2_CO_3_ were the carbonates (calcite, gaylussite, Ht, Hc, and Mc) and the strength-giving phase (C-A-S-H). Both increasing the CRCF dosage and enhancing the Na_2_CO_3_ can promote the formation of carbonates, except for gaylussite (a transient phase).
(3) Incorporating CRCF to a significant degree promoted the compressive strength development of AAS mortars, which can be further improved by increasing the amount of Na_2_CO_3_. The highest compressive strength (39.2 MPa) can be observed in AAS mortars with 6% Na_2_O-E and 15% CRCF.

Based on the above data, CRCF worked as an auxiliary activator to accelerate the reaction kinetics and exhibited the potential to be effectively applied in the Na_2_CO_3_-activated GBFS. Considering the compressive strength results, the preliminarily recommended optimal composite proportion of GBFS:CRCF was 85:15, and the Na_2_O-E of Na_2_CO_3_ was 6% by the mass of binders. A feasible method to further improve the strength development of mortars and applications in structural concrete will continue to be explored in future works.

## Figures and Tables

**Table 1:** Chemical compositions of GBFS and CRCF.

Raw Materials	Chemical Composition (wt.%)						
	SiO_2_	Al_2_O_3_	CaO	MgO	Fe_2_O_3_	Na_2_O	SO_3_
GBFS	27.2	15.5	44.8	8.0	0.3	0.3	2.3
CRCF	28. 4	7.0	56.1	1.4	3.0	0.3	2.1

**Table 2:** Mix proportions of mortars.

Code	GBFS/g	CRCF/g	Sand/g	Na_2_CO_3_/g	Na_2_O-E/%	Water/g
CR0S5	344.8	0	500	29.5	5	155.2
CR5S5	327.6	17.2	500	29.5	5	155.2
CR10S5	310.3	34.5	500	29.5	5	155.2
CR15S5	293.1	51.7	500	29.5	5	155.2
CR20S5	274.8	70.0	500	29.5	5	155.2
CR30S5	241.4	103.4	500	29.5	5	155.2
CR15S3	293.1	51.7	500	17.7	3	155.2
CR15S4	293.1	51.7	500	23.6	4	155.2
CR15S6	293.1	51.7	500	35.4	6	155.2
CR15S7	293.1	51.7	500	41.3	7	155.2

**Table 3:** Mass loss (%) of paste samples at different temperature ranges.

Mix	Dehydration of Bound Water		Dehydroxylaztion of Ht		Decarbonation of Ht and Low-Crystallinity Carbonates		Decarbonation of Calcite	
	40–250 °C		300–400 °C		450–600 °C		600–700 °C	
	3 d	28 d	3 d	28 d	3 d	28 d	3 d	28 d
CR0S5	0.94	1.66	0.20	0.84	0.92	0.89	0.73	1.60
CR5S5	8.26	9.65	1.75	2.10	3.58	3.36	1.40	1.74
CR15S5	8.46	10.09	1.98	2.42	4.35	3.39	1.34	2.45
CR30S5	9.40	9.60	2.36	2.47	4.85	4.02	1.41	2.66
CR15S3	6.21	7.06	1.45	1.66	4.74	3.42	0.83	1.65
CR15S7	10.10	10.16	2.27	2.47	3.70	3.15	1.83	2.55
